# Neuropsychologic Profiles and Cerebral Glucose Metabolism in Neurocognitive Long COVID Syndrome

**DOI:** 10.2967/jnumed.121.262677

**Published:** 2022-07

**Authors:** Andrea Dressing, Tobias Bormann, Ganna Blazhenets, Nils Schroeter, Lea I. Walter, Johannes Thurow, Dietrich August, Hanna Hilger, Katarina Stete, Kathrin Gerstacker, Susan Arndt, Alexander Rau, Horst Urbach, Siegbert Rieg, Dirk Wagner, Cornelius Weiller, Philipp T. Meyer, Jonas A. Hosp

**Affiliations:** 1 Department of Neurology and Clinical Neuroscience, Medical Center – University of Freiburg, Faculty of Medicine, University of Freiburg, Freiburg, Germany;; 2 Freiburg Brain Imaging Center, Medical Center – University of Freiburg, Faculty of Medicine, University of Freiburg, Freiburg, Germany;; 3 Department of Nuclear Medicine, Medical Center – University of Freiburg, Faculty of Medicine, University of Freiburg, Freiburg, Germany;; 4 Division of Infectious Diseases, Department of Medicine II, Medical Center – University of Freiburg, Faculty of Medicine, University of Freiburg, Freiburg, Germany;; 5 Department of Otorhinolaryngology – Head and Neck Surgery, Medical Center – University of Freiburg, Faculty of Medicine, University of Freiburg; and; 6 Department of Neuroradiology, Medical Center – University of Freiburg, Faculty of Medicine, University of Freiburg, Freiburg, Germany

**Keywords:** long COVID syndrome, cognition, fatigue, ^18^F-FDG PET, Montreal Cognitive Assessment

## Abstract

During the coronavirus disease 2019 (COVID-19) pandemic, Long COVID syndrome, which impairs patients through cognitive deficits, fatigue, and exhaustion, has become increasingly relevant. Its underlying pathophysiology, however, is unknown. In this study, we assessed cognitive profiles and regional cerebral glucose metabolism as a biomarker of neuronal function in outpatients with long-term neurocognitive symptoms after COVID-19. **Methods:** Outpatients seeking neurologic counseling with neurocognitive symptoms persisting for more than 3 mo after polymerase chain reaction (PCR)–confirmed COVID-19 were included prospectively between June 16, 2020, and January 29, 2021. Patients (*n* = 31; age, 53.6 ± 2.0 y) in the long-term phase after COVID-19 (202 ± 58 d after positive PCR) were assessed with a neuropsychologic test battery. Cerebral ^18^F-FDG PET imaging was performed in 14 of 31 patients. **Results:** Patients self-reported impaired attention, memory, and multitasking abilities (31/31), word-finding difficulties (27/31), and fatigue (24/31). Twelve of 31 patients could not return to the previous level of independence/employment. For all cognitive domains, average group results of the neuropsychologic test battery showed no impairment, but deficits (*z* score < −1.5) were present on a single-patient level mainly in the domain of visual memory (in 7/31; other domains ≤ 2/31). Mean Montreal Cognitive Assessment performance (27/30 points) was above the cutoff value for detection of cognitive impairment (<26 points), although 9 of 31 patients performed slightly below this level (23–25 points). In the subgroup of patients who underwent ^18^F-FDG PET, we found no significant changes of regional cerebral glucose metabolism. **Conclusion:** Long COVID patients self-report uniform symptoms hampering their ability to work in a relevant fraction. However, cognitive testing showed minor impairments only on a single-patient level approximately 6 mo after the infection, whereas functional imaging revealed no distinct pathologic changes. This clearly deviates from previous findings in subacute COVID-19 patients, suggesting that underlying neuronal causes are different and possibly related to the high prevalence of fatigue.

As the coronavirus disease 2019 (COVID-19) pandemic proceeds, the long-term consequences such as chronic neurocognitive symptoms after infection with the severe acute respiratory syndrome coronavirus 2 (SARS-CoV-2) are an increasingly recognized problem. A multitude of previously healthy patients self-report symptoms such as brain-fog, memory loss (18%–40%), attentional problems (16%–34%), and fatigue (60%–70%) months after the acute infection has long subsided (1–6). The label “long COVID syndrome” has recently been established for these symptoms in the aftermath of an acute SARS-CoV-2 infection (7); however, the underlying pathophysiology remains unclear.

We described impaired cognitive functions associated with frontoparietal hypometabolism (indicating cortical dysfunction) on ^18^F-FDG PET (8) in COVID-19 patients approximately 1 mo after the acute infection. When voxelwise principal components analysis is used, a COVID-19–related spatial covariance pattern has emerged, the expression of which tightly correlates with performance in the Montreal Cognitive Assessment (MoCA). In a subgroup of these patients, a long-term follow-up (6–7 mo after infection) revealed a substantial but still incomplete recovery of cognitive deficits and cortical dysfunction on ^18^F-FDG PET (9). Likewise, a predominantly frontal cortical hypometabolism, which improved during follow-up after 6 mo, was detected in patients with COVID-19–related encephalopathy (10). Deviating from these findings, regional hypometabolism of limbic and paralimbic regions extending to the brain stem and cerebellum (11) or hypometabolism of the right parahippocampal gyrus and thalamus (12) has been described in COVID-19 patients examined at 3–4 mo after symptom onset.

Postmortem neuropathologic examinations in COVID-19 patients revealed pronounced glial activation and infiltration by cytotoxic T lymphocytes in the brain stem and cerebellum (13), likely caused by a systemic inflammatory response or a cytokine release (14). Because the cortical gray matter is largely unaffected by inflammatory changes (8*,*^13^), reduction of cortical glucose metabolism in early subacute patients (8–10) might be caused by a functional decoupling from afferents, which is in line with recovery of cognitive deficits and cortical metabolism in long-term follow-up investigations (9*,*^10^*,*^15^).

Thus, the question arises whether alterations in cerebral glucose metabolism are also present in patients with long COVID syndrome as a potential pathophysiologic correlate of the neurocognitive symptoms. We present data from a prospective cohort of outpatients about 6 mo after SARS-CoV-2 infection who self-reported persistent subjective neurocognitive symptoms. Cognitive performance and cerebral ^18^F-FDG PET were assessed to objectify subjective symptoms and to investigate possible similarities to previously observed changes in early subacute patients.

## MATERIALS AND METHODS

### Participants

We report data from a monocentric, prospective cohort of 31 patients (age, 53.6 ± 12.0 y; 11 men, 20 women) who were admitted to the outpatient clinic of the Department of Neurology and Clinical Neuroscience of the University Hospital Freiburg between June 16, 2020, and January 29, 2021 due to lasting neurocognitive symptoms in the chronic phase (>3 mo) after COVID-19. Inclusion criteria were a history of reverse transcription polymerase chain reaction (rt-PCR)–confirmed SARS-CoV-2 infection, presence of new subjective neurocognitive symptoms persisting for longer than 3 mo after positive rt-PCR, and age > 18 y. Exclusion criteria were any preexisting neurodegenerative disorders. One patient refused to participate. Detailed demographic data are provided in Supplemental Table 1 (supplemental materials are available at http://jnm.snmjournals.org).

Importantly, the current long COVID cohort shares no overlap with previous studies on COVID-19 from our group (8*,*^9^). In these, subacute inpatients (3–4 wk post-COVID) were screened independently from subjective complaints and included if they met inclusion criteria (most importantly, at least 2 new neurologic symptoms to qualify for PET) (8). A subset of 8 patients was furthermore eligible for a follow-up (9). In contrast, the present cohort results from self-referral because of new neurocognitive symptoms, which may not necessarily be verified by further examinations (see the section “Cognitive Functions”).

The present study was approved by the local ethics committee of the University Medical Center Freiburg (EK 211/20) and complies with the Helsinki Declaration of 1975, as revised in 2008. Written informed consent was obtained from all patients.

### General Examination

General neurologic deficits were examined in a complete neurologic assessment by a board-certified neurologist (>5 y of training). The degree of actual disability was graded as follows: 0, no relevant restrictions; 1, relevant restrictions but able to work; 2, reduction of work quota necessary; 3, inability to work or restriction of daily life activities. Disease severity during the acute stage was scored according to a modified version of the German definitions (16): 1, no signs of pneumonia; 2, pneumonia, outpatient treatment; 3, pneumonia, inpatient treatment; 4, acute respiratory distress syndrome, endotracheal ventilation in intensive care unit. A subgroup of 6 patients received structural MRI (supplemental methods).

### Cognitive Functions

All patients were examined with a 50-min cognitive battery administered in German (native language) in a set order by a trained neuropsychologist. The neuropsychologic test battery comprised the Hopkins Verbal Learning Test-Revised (HVLT (17)), Brief Visuospatial Memory Test-Revised (BVMT-R (18)), Digit Span forward/reverse (19), Trail Making Test part A/B (20), Color-Word Interference Test (FWIT (21), Symbol-Digit Modalities Test (SDMT (22)), and a semantic and letter fluency test (23). Individual raw scores were z-transformed based on the normative sample as reported in the manuals. Results were stratified by age and education where available. In the case of the FWIT, raw scores were assigned a T score, which then was transformed into a *z* score. *z* scores for each domain and a composite *z* score that represents overall cognitive functions of the patients were calculated by averaging the z sores based on Lazar et al. (24) with minor adjustments. The threshold for impaired performance was defined as 1.5 SDs below the normative mean (24). Additionally, the MoCA (version 7.1, www.mocatest.org (25)) was applied (maximum achievable score = 30, higher scores indicating better performance). The cutoff score for cognitive impairment was defined as performance below 26 (*25*). A correction for years of education (YoE) was performed (+1 point in case of ≤ 12 YoE). Fatigue was assessed using the Würzburg Fatigue Inventory in Multiple Sclerosis (WEIMuS (26)), a self-rating questionnaire for symptoms of physical and cognitive fatigue. In addition, the Geriatric Depression Scale-15 (GDS (27)) was included. Scores for the MoCA, fatigue, and the GDS were not included in the composite score.

### ^18^F-FDG PET Imaging

Cerebral ^18^F-FDG PET was recommended to all patients on the basis of clinical indication for diagnosis of persistent unexplained cognitive impairment (including the exclusion of other causes) based on previous reports on altered cerebral glucose metabolism in COVID-19 patients (8*,*^10^–^12^). Imaging was performed in 14 of 31 patients on average 197.9 ± 61.1 d after manifestation of COVID-19 as indicated by the first positive PCR. PET scans (10-min duration) were acquired on a fully digital Vereos PET/CT scanner (Philips Healthcare) 50 min after intravenous injection of 211 ± 9 MBq of ^18^F-FDG under euglycemic conditions at rest (eyes open, reduced ambient noise). All individual scans were read as part of the clinical routine by 2 experienced nuclear medicine physicians (>20 and 5 y of experience in brain PET) using highly standardized displays of 30 transaxial ^18^F-FDG PET slices (hot metal color scale; maximum and minimum thresholds set to 1.8 [100%] and 0.09 [5%], respectively, after voxelwise data normalization to mean uptake in brain parenchyma) and voxel-based statistical analyses using 3-dimensional stereotactic surface projections (3D-SSP/Neurostat (28)) and appropriate age-matched controls.

Group analyses were performed as previously described (8): after spatial normalization and smoothing (isotropic gaussian kernel, 10 mm in full width at half maximum), the pattern expression score (PES) of the previously established COVID-19–related spatial covariance pattern was derived by the topographic profile rating algorithm, reflecting the expression of the established pattern in each individual’s data. For statistical comparison, we also assessed the PES of the COVID-19–related covariance pattern in control patients (*n* = 45; age, 63.0 ± 9.1 y; age range, 50–85 y; 27 men, 18 women) scanned under identical conditions (8). As confirmatory analysis, a voxelwise analysis of covariance (ANCOVA) with age and sex as covariates was calculated with statistical parametric mapping (SPM) for comparison of long COVID and control patients. For count rate normalization, we used proportional scaling of each individual’s ^18^F-FDG PET data to the mean uptake in a brain parenchyma mask (SPM tissue probability map, white and gray matter probability > 50% excluding cerebrospinal fluid with probability > 30%). A false-discovery rate (FDR)–corrected *P* < 0.05 was used as a statistical threshold. The correlation between mean *z* scores of the domains (attention, executive function, processing speed, verbal and visual memory), composite *z* score, MoCA, WEIMuS cognitive and physical fatigue scores, and voxelwise ^18^F-FDG uptake was analyzed by SPM-based regression analyses. FDR-corrected *P* < 0.05 and uncorrected *P* < 0.005 (cluster size > 30 voxels) were used as statistical thresholds. All processing steps were implemented with an in-house pipeline using MATLAB (The MathWorks, Inc.) and SPM (SPM12; The Wellcome Centre for Human Neuroimaging, UCL Queen Square Institute of Neurology) software.

### Statistical Analysis

Statistical analyses were performed using SPSS Statistics, version 27 (IBM) and R (https://www.R-project.org/). Shapiro–Wilk and Kolmogorov–Smirnov tests were used to confirm normal distribution. Correlations between demographic and clinical data and neurocognitive test scores were exploratorily assessed with the Spearman rank correlation test. For group comparisons of neurocognitive test scores, 1-sample *t* or Mann–Whitney *U* tests were performed. The group difference of the PES of long COVID patients and control patients was tested with an ANCOVA including age and sex as covariates. The strength of the relationship between the PES of the COVID-19–related covariance pattern and the results from cognitive assessments was estimated with a Spearman rank partial correlation test adjusted for the patient’s age.

## RESULTS

The neurologic examination (202.3 ± 57.5 d after first positive COVID-19 PCR) revealed no focal deficit related to SARS-CoV-2 infection. On the contrary, all patients complained about difficulties in attention, memory, and multitasking abilities. Moreover, 24 of 31 (77%) complained about fatigue. Three of 31 (10%) patients reduced their work quota due to these symptoms; 9 of 31 (29%) patients were unable to work or restricted their activities of daily living at the time of examination. Actual disability was significantly correlated with severity of initial disease (*R* = 0.38; *P* = 0.03). Basic clinical data are summarized in Supplemental Table 2.

Six of 31 patients underwent cerebral MRI (4 with contrast enhancement). On visual assessment, microembolic subacute cortical infarction was observed in the left occipital lobe in 1 patient (65-y-old man), and slight microangiopathic changes corresponding to Fazekas 2 were present in a 61-y-old female patient. No other structural changes, and in particular no sign of atrophy, acute encephalitis, or leptomeningeal enhancement, were found.

### Cognitive Functions

The mean *z* scores of verbal and visual memory domains and composite *z* score were not significantly different from zero (all *P* > 0.1). The mean *z* scores for executive functions (*P* < 0.05), attention (*P* < 0.01), and processing speed (*P* < 0.01) were even higher than zero and, in total, almost half of the patients (*n* = 15, 49%) were completely unimpaired in the neurocognitive test battery (Supplemental Table 3). However, some patients exhibited mild to moderate impairments in single domains: the most frequently impaired domain was visual memory (7/31 [23%] patients; other domains ≤ 2/31 [≤7%]). Impaired individual tests on a single-subject level were most frequently observed for verbal and visual memory tests (number of impaired patients 3–7 [10%–23%] and 4–8 [13%–26%], respectively; Supplemental Table 3).

Although the mean group MoCA performance (26.6 ± 2.2 points) was above the cutoff (25), mild impairment was detected in 9 patients (29%; range, 23–25 points). The greatest variance was observed for the recall task of the MoCA (3.2 ± 1.6 points, 16/31 patients scoring below 4 points). The group of patients with an impaired MoCA test did not differ in terms of age or delay between infection and examination from the rest of the cohort (both *P* > 0.5).

On a self-rating questionnaire, 61% (*n* = 19) revealed overall symptoms of fatigue. On a subscore level, 67% (*n* = 21) were above the cutoff for cognitive fatigue and 42% (*n* = 13) were above the cutoff for physical fatigue (Supplemental Table 4). The GDS (3.9 ± 2.6) indicated no relevant level of depression in the present patient cohort; only 4 individuals slightly exceeded the cutoff value (range, 8–10 points) indicating mild depressive symptoms (Supplemental Table 4) (27).

Performance on MoCA was correlated with the composite *z* score of the neurocognitive test battery (*R* = 0.53; *P* < 0.05). In turn, self-rated fatigue (WEIMuS sum score) correlated significantly with self-rated depression (GDS, albeit in a subclinical range; *R* = 0.61; *P* < 0.001). MoCA test scores and the overall composite *z* score did not correlate with self-rated depression or any aspect of self-rated fatigue. Except for positive correlations of initial disease severity with physical fatigue (*R* = 0.37; *P* < 0.05), clinical parameters such as disease severity and degree of actual disability did not correlate with performance on cognitive and other tests.

### ^18^F-FDG PET Imaging

**FIGURE 1.**
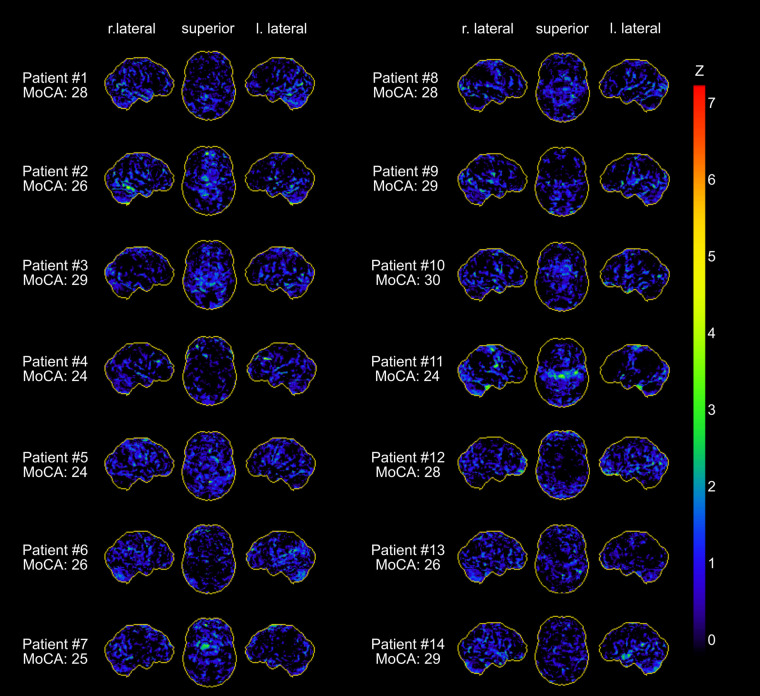
Individual results of voxelwise statistical analysis of ^18^F-FDG PET data with NeuroSTAT/3D-SSP (stereotactic surface projection). Shown are lateral and superior views of brain. Metabolic deficits compared with age-matched control subjects are color-coded as *z* scores. Z = *z* score.

Patients undergoing ^18^F-FDG PET did not differ from those who did not in terms of epidemiologic variables (age, sex) or results of the neuropsychologic test battery (all *P* > 0.05, Supplemental Tables 1–4). Clinical routine assessments of each patient’s ^18^F-FDG PET scan revealed no distinct pathologic findings (Fig. 1). In particular, none of the patients exhibited a frontoparietal predominant hypometabolic pattern previously described in subacute COVID-19 inpatients (8). Likewise, PET scans suggested no alternative diagnoses (e.g., encephalitis, neurodegenerative dementia) in any case.

**FIGURE 2.**
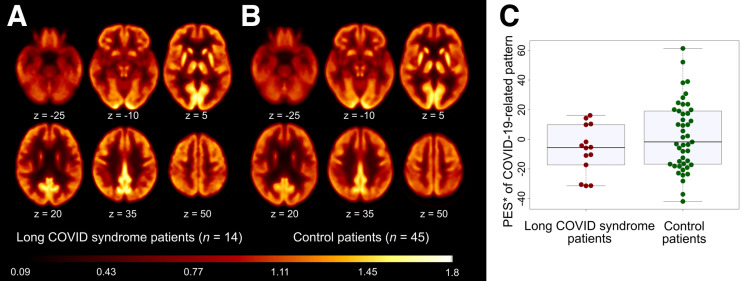
^18^F-FDG PET in patients with long COVID syndrome. (A and B) Transaxial sections of group averaged, spatially normalized ^18^F-FDG PET scans in patients with long COVID syndrome (A) and control patients (B). (C) The pattern expression score (PES; *adjusted for age and sex, for illustration purposes) of previously established COVID-19–related spatial covariance pattern was not significantly different between patients with long COVID syndrome and control patients. Box plots (gray), as well as individual values for COVID-19 patients (red) and the control cohort (green), are displayed.

Group-averaged ^18^F-FDG PET scans in long COVID and control patients are shown in Figure 2. None of the patients expressed the previously established COVID-19–related spatial covariance pattern, with individual PES ranging from −7 to −60. There was no significant group difference in PES between long COVID patients (−36.7 ± 17.3) and control patients (−11.3 ± 29.2) after adjustment for age and sex (ANCOVA, factor group: *P* = 0.14).

In contrast to our previous studies in COVID-19 inpatients with novel neurologic symptoms in the subacute stage (8) and at follow-up (9), no significant relationship between MoCA and PES was found (R = −0.17, *P* > 0.5). There was also no significant correlation of PES with fatigue, composite or domains *z* scores (all *P* > 0.5). Confirmatory voxelwise SPM analyses yielded no regions with significantly (FDR-corrected *P* < 0.05) different glucose metabolism (neither hyper- nor hypometabolism) in long COVID patients compared with control patients. Moreover, no association to MoCA, domain *z* scores, composite *z* score, or WEIMuS fatigue scores were found by voxelwise regression analyses (FDR-corrected *P* < 0.05). No relevant findings were observed at an exploratory statistical threshold of uncorrected *P* < 0.005.

## DISCUSSION

The present study reports a prospective assessment of 31 patients self-presenting to our outpatient clinic because of neurocognitive symptoms more than 6 mo after a SARS-CoV-2 infection with long COVID syndrome. Although 39% of patients report a relevant disability at work and everyday life due to these symptoms, an exhaustive assessment including a detailed cognitive battery showed only mild impairment in individual patients, and cerebral ^18^F-FDG PET failed to reveal a distinct pathologic signature.

Cognitive profiles in our sample revealed an overall normal to higher-than-normal performance in all cognitive domains (verbal memory, visual memory, processing speed, attention, executive function) and on MoCA (average score 27/30, cutoff value for detection of any cognitive impairment < 26/30 (*25*)). However, impairments were present on a single-patient level, especially in the domain of visual memory (Supplemental Table 3). Furthermore, 9 of 31 (29%) patients performed below the MoCA cutoff value, indicating mild cognitive impairments.

These results indicate that in some patients with long COVID, discrete neurocognitive impairments may be present, which is in line with findings from other studies: deficits in verbal and visual memory, executive functions, verbal fluency, attention, and language were reported 6–9 mo after the infection, which were correlated in their expression with the initial degree of severity (29). Another study reported mild deficits in episodic memory function (up to 6 mo after the infection) and vigilance and motivation deficits (up to 9 mo after the infection); the deficits normalized after the corresponding period of time (15). This, in combination with the longitudinal assessment of COVID-19 patients from our group (8*,*^9^), suggests that the cognitive deficits are subject to a dynamic process, which might also explain why most patients are cognitively unimpaired in the present long-term study.

It has to be noted that a rather liberal threshold for definition of impaired cognition was used (1.5 SD < normative mean). This threshold corresponds to a 1-sided *P* value of about 0.07, which increases the risk of false-positive results and is only slightly below the frequency of impaired observations on detailed tests (Supplemental Table 3: 45/496, 9.1%). That impaired scales accumulated in verbal and visual memory tests is not surprising, as the Hopkins verbal learning test and BVMT are especially challenging and susceptible for attentional fluctuations (24*,*^28^). Such fluctuations may also explain why more patients showed impairments in the recognition (i.e., 8/31) when compared with the delayed recall part (i.e., 4/31) of the BVMT—although the latter usually detects deficits with a higher sensitivity (30). Although the comprehensive neuropsychologic test battery indicated slight deficits at the level of individual patients, affection of MoCA performance seemed to be more severely pronounced. This could also be explained by motivational deficits, attentional fluctuations and exhaustion as the MoCA was performed at the end of the test battery.

Previous studies in subacute COVID-19 patients showed deficits in executive and attentional functions, memory, and visuospatial functions that point to a cortical dysfunction with a frontoparietal emphasis (4*,*^29^*,*^31^). As a correlate of impaired cognitive functions, we recently described a predominantly frontoparietal cortical hypometabolism on ^18^F-FDG PET in subacute COVID-19 patients (8*,*^9^). Thus, we also performed ^18^F-FDG PET in the present sample of patients with neurocognitive long COVID syndrome to objectify changes of regional neuronal function by an approach that is independent of the patients’ test compliance and can be analyzed completely observer-independent. Individual ^18^F-FDG PET reads did not reveal any distinct pathologic finding, including possible alternative diagnoses, in any of the patients. We also analyzed the PES of the previously established COVID-19–related metabolic covariance pattern. However, whereas this pattern tightly correlated to MoCA performance and was still elevated at trend level compared with control patients at follow-up in our studies in COVID-19 inpatients (at the subacute stage and 6 mo later) (8*,*^9^), none of the patients with long COVID syndrome exhibited this pattern. Notably, this also includes 4 of 14 patients showing impaired performance on MoCA who underwent ^18^F-FDG PET. To exclude the possibility that the COVID-19–related metabolic covariance pattern established in subacute inpatients is simply not appropriate for patients with long COVID syndrome, we also conducted a conventional SPM group analysis, which, again, showed no pathologic finding. Even for a subgroup of patients with abnormal MoCA scores (*n* = 4), we did not find any relevant differences in glucose metabolism compared with the remaining patients or the control cohort (voxelwise SPM group analysis, exploratory threshold of *P* < 0.005, data not shown). Although we cannot exclude the possibility that long COVID–associated changes of neuronal activity are too subtle to be captured by an ^18^F-FDG PET group analysis, we consider this unlikely. Indeed, ^18^F-FDG PET is a well-established marker of neuronal dysfunction for prodromal stages of neurologic diseases of similar cognitive impact. Thus, together with the in large parts unimpaired cognitive battery across the entire group of patients, it appears reasonable that factors other than the cortical hypometabolism observed in patients during the early subacute stage after an infection (8) contribute to the symptoms in neurocognitive long COVID syndrome.

Our results have to be compared with other recent studies that used cerebral ^18^F-FDG PET for the assessment of COVID-19–associated metabolic changes. A frontal and, to a lesser extent, temporoparietal cortical hypometabolism, which improved during follow-up at 1 and 6 mo, was detected by Kas et al. (10), which is in line with our observations (8*,*^9^). Of note, different from our previous cohort (8*,*^9^) patients included in the aforementioned study (10) suffered from COVID-19–related encephalopathy including delirium, seizures, myocloni, and focal neurologic signs, whereas such severe symptoms were absent in the patients of our subacute cohort (8*,*^9^). Clearly deviating from those studies and the present study, a profile of hypometabolism in limbic and paralimbic regions extending to the brain stem and cerebellum was reported for patients with putative long COVID (including decreasing glucose metabolism of the right temporal lobe with longer time after first COVID-19 symptoms) (11). Factors such as pooling of variable time points of examination (about 1–5 mo after COVID-19, on average 96 ± 31 d) and the use of cortical regions for count rate normalization of PET data may have contributed to these discordant findings that are also counterintuitive regarding recovery from COVID-related cognitive deficits in longitudinal investigations (9*,*^15^) (a detailed discussion appears in Meyer et al. (32)). Sollini et al. (12) described a hypometabolism particularly of the right parahippocampal gyrus and thalamus in long COVID patients examined at about 3–4 mo after symptom onset. Again, technical factors may explain different findings (e.g., extraction of brain scans from whole-body examination, which may yield inferior data quality if whole-body PET acquisition parameters are not matched to brain acquisitions; retrospective use of brain images of oncologic patients as control data, which limits standardization of behavior/sensory input during ^18^F-FDG uptake; and liberal statistical thresholds such as *P* < 0.005 on the voxel level). Finally, a thorough qualitative and quantitative assessment of cognitive profiles and correlation to changes in cerebral glucose metabolism was not pursued by other studies (10–12), which underlines the particular value of the present work.

The lack of significant findings on ^18^F-FDG PET and only mild impairments on neuropsychologic testing is in contrast to the severe and lasting disability reported by the patients (e.g., cognitive symptoms, inability to work). Moreover, neither MoCA performance nor the composite *z* score of the neurocognitive test battery correlated with disability. On the other hand and in line with other reports in long COVID (33*,*^34^), fatigue was particularly prevalent in our cohort (61%, WEIMuS sumscore). Fatigue is a common sequela of systemic viral infections (35*,*^36^) and systemic inflammatory diseases (37) and has been related to immune dysregulation processes (38*,*^39^) as in the systemic inflammatory response and cytokine release (14) in COVID-19. Fatigue has also been linked to the myalgic encephalomyelitis/chronic fatigue syndrome (5) in long COVID, which is characterized by functional impairment (e.g., disability to work) in a considerable number of patients (40). Taken together, it is tempting to speculate that the pathophysiologic background of self-reported cognitive symptoms, disability, and even mild impairments in the neuropsychologic test battery in single patients is primarily caused by fatigue.

As a limitation of the present study, only patients self-presenting with long-lasting symptoms were included in our cohort, thereby potentially presenting a small subgroup of COVID-19 patients. However, deficits reported in our cohort are corroborated by the rate of previously reported deficits (2*,*^3^), and the fraction of patients that were still unable to work 3–4 mo after infection reported previously (2) is in accordance with our cohort (i.e., 32% vs. 39%). As an inherent problem of studies like ours, no data are available concerning the premorbid cognitive and neuropsychologic status of the patients. Thus, we cannot comment on a possible particular vulnerability or preexisting deficits, which is of particular interest if the detected impairment is small and inconsistent. Furthermore, the number of subjects is relatively small, which precluded in-depth multivariate statistical analyses and limited the possibility to make reliable statements about the frequency of cognitive deficits in long COVID patients. For instance, in contrast to the general observation that male sex is a risk factor for severe disease courses in COVID-19 (*41*), female sex seems to be slightly overrepresented in the present study. Longitudinal studies are needed to define the prognosis of neurocognitive symptoms in patients with long COVID syndrome. In this regard, the lack of long-lasting alterations of cerebral functioning on ^18^F-FDG PET would be compatible with a favorable outcome.

## DISCLOSURE

Philipp T. Meyer received honoraria for lectures and consulting by GE and Philips. Horst Urbach received honoraria for lectures from Bracco, Bayer, Union Chimique Belge (UCB) pharma, Eisai, and Stryker. Nils Schroeter and Andrea Dressing were supported by the Berta-Ottenstein-Program for Clinician Scientists, Faculty of Medicine, University of Freiburg. No other potential conflict of interest relevant to this article was reported.

## Notes

KEY POINTS **QUESTION:** Long-term neurocognitive symptoms after COVID-19 are an increasingly recognized problem—is the underlying pathophysiology a residual state of cortical dysfunction detected in subacute patients? **PERTINENT FINDINGS:** In patients (*n* = 31) in the long-term phase after COVID-19 (202.3 ± 57.5 d after positive PCR) with self-reported symptoms of long COVID, an exhaustive neuropsychologic test battery revealed slight impairments only in individual cases, whereas fatigue was highly prevalent. Cerebral ^18^F-FDG PET failed to reveal a distinct pathologic signature in the subgroup of patients undergoing ^18^F-FDG PET (*n* = 14). **IMPLICATIONS FOR PATIENT CARE:** The findings deviate from previous reports in patients in the early subacute stage of COVID-19 and suggest that underlying causes of long COVID might be related to fatigue but not to persistent cortical dysfunction.
